# Changes in Consumers’ Food Practices during the COVID-19 Lockdown, Implications for Diet Quality and the Food System: A Cross-Continental Comparison

**DOI:** 10.3390/nu13010020

**Published:** 2020-12-23

**Authors:** Blain Murphy, Tony Benson, Amanda McCloat, Elaine Mooney, Chris Elliott, Moira Dean, Fiona Lavelle

**Affiliations:** 1 Institute for Global Food Security, School of Biological Sciences, Queen’s University Belfast, Belfast BT9 5DL, UK; B.murphy@qub.ac.uk (B.M.); t.benson@qub.ac.uk (T.B.); chris.elliott@qub.ac.uk (C.E.); moira.dean@qub.ac.uk (M.D.); 2 Department of Home Economics, St. Angela’s College, F91 C634 Sligo, Ireland; amccloat@stangelas.nuigalway.ie (A.M.); emooney@stangelas.nuigalway.ie (E.M.)

**Keywords:** COVID-19, food practices, cooking, diet quality, food skills, food systems, survey, consumer, health, cross-continental

## Abstract

COVID-19 has led to dramatic societal changes. Differing movement restrictions across countries have affected changes in consumers’ food practices, with a potentially detrimental impact on their health and food systems. To investigate this, this research explored changes in consumers’ food practices during the initial COVID-19 phase and assessed the impact of location on these changes. A sample of 2360 adults from three continents (Island of Ireland (IOI), Great Britain (GB), United States (USA), and New Zealand (NZ)) were recruited for a cross-sectional online survey (May–June 2020). Participants completed questions in relation to their cooking and food practices, diet quality, and COVID-19 food-related practices. Significant changes in consumers’ food practices during the pandemic were seen within and between regions, with fewer cooking practices changes found in the USA. Food practices, which may put added pressure on the food system, such as bulk buying, were seen across all regions. To prevent this, organisational food practices, including planning ahead, should be emphasized. Additionally, while positive cooking-related practices and increases in fruit and vegetable intake were found, an increase in saturated fat intake was also seen. With the additional pressure on individuals’ physical and mental health, the essentiality of maintaining a balanced diet should be promoted.

## 1. Introduction

COVID-19 is an unprecedented global pandemic. As of the 26th October 2020, there were >43,000,000 reported cases and more than 1,100,000 fatalities worldwide [1]. Over half of the world’s population is, or has been, under some form of social distancing or lockdown in an attempt to contain the health crisis [2]. This has led to a number of dramatic societal changes including the closure of businesses [2] and a shift to a ‘working from home’ business model [3].These societal changes have also led to alterations in individual’s food practices. The closure of businesses and the uncertainty of the current and future situation led to an increase in panic buying among consumers [4]. Furthermore, the closure of businesses, including hospitality services, reportedly resulted in a shift of consumption patterns to meals prepared and consumed in the home [5].

The lack of options and increased time in the home environment, through lockdowns and working from home, may have presented an opportunity to overcome previously often cited barriers to cooking, such as a lack of time and the effort required for meal preparation [6]. However, the rapid shifts in societal food practices may lead to excessive pressure on food supply chains that may be vulnerable to these “Demand-side shocks” [5]. As the pandemic continues and countries implement different levels of social distancing and lockdowns, food supply chains may build resiliency for these “Demand-side shocks” through purchase limits and rationing mechanisms, but may need to focus more on the “Supply-side shocks”, such as labor shortages and disruptions to transport networks [5]. To enable food supply chain resiliency, it is essential to understand changes in consumer food practices in lockdown circumstances to manage the current situation and to prepare for future emergency situations.

Food skills or practices, such as meal planning and shopping with a grocery list, have been associated with positive dietary patterns and diet quality in Irish, UK, and Australasia samples [7,8]. Furthermore, food skills are a contributing factor to Food Agency, which, in turn, is associated with enhanced dietary intake and positive cooking behaviours in a USA sample [9]. Additionally, the consumption of home cooked meals has been associated with several positive health outcomes such as improved weight maintenance, and a normal Body Mass Index (BMI) and percentage body fat [10,11]. Specific foods that are shown to increase or decrease with home cooking have their own associations with health outcomes, such as increased consumption of fruit and vegetables with positive mental well-being [12], increases in these are associated with meal preparation [13]. Whereas sweets, snacks, and fast food, typically reduced with increases in cooking [10], are associated with perceived stress and depressive symptoms [14]. However, there has been a reported concern that there is a decline in home cooking in the above mentioned regions [15,16,17,18]. All have seen an increase in the use of convenience foods, although the USA and GB may be seen as higher convenience food consumers, with Ireland and New Zealand narrowing the gap [19,20,21,22]. In light of this, numerous interventions have been undertaken to try increase home cooking [7,23,24]. With the reported anecdotal changes in cooking and food practices [5], it is important to understand what actual changes are occurring from these dramatic societal changes (for example additional time), as these can have an influence on health outcomes that are being exasperated during COVID-19.

In order to provide a cross-continental comparison, four countries (New Zealand, USA, Great Britain, and the Island of Ireland) were chosen as comparator countries as they have all reported concerns around a decline in home cooking and implemented interventions to try increase cooking and similar socio-economic profiles, but differed in their COVID-19 management policies. The countries and governments adopted various strategies and approaches to curtailing the spread of the virus, reflecting differences in resources, cultures, law, and phases of the epidemic [25]. New Zealand is seen as a relative success case in controlling and eliminating the virus, which began implementing planning for the pandemic as early as February, when the first case was reported, through preparing hospitals and border controls [26]. When community transmission was apparent, due to insufficient testing and tracing capacity, the government implemented a stringent lockdown on March 26th [26]. New Zealand currently has a low case count and one of the lowest global mortality rates [26]. Meanwhile, the USA had within country differences in their approach to managing COVID-19 [25]. The first case in the USA was reported in January and, without a centralized approach to the management, the USA quickly became the global epicenter for the virus [27]. The decentralized approach left state and local authorities to implement and enforce social distancing measures [28]. Data highlighted that in areas where social distancing occurred; there was a reduction in case growth [28]. In Europe, there were many different approaches taken, depending on the country and phase of the epidemic. Two regions, Ireland and Great Britain, which have amongst the lowest hospital bed capacity in Europe [29], implemented different approaches at the beginning of the COVID-19 pandemic. Although technically the UK implemented a national lockdown before Ireland [30,31], Ireland had been implementing a delay phase before this, including the closure of schools, universities and public houses. From all these examples, it is important to understand whether government’s approaches, either hard-line or delayed, influenced the level of changes seen in individual’s food practices and cooking behaviours, due to the level of restrictions. For example, if a region was not restricted to working from home, their cooking behaviours may not have differed to their norm.

Therefore, in light of the impact that changes in consumer’s food practices may have on both food systems and their own health, this research aimed to investigate the changes in consumers’ food practices during the initial phase of the COVID-19 pandemic. Furthermore, this study aimed to investigate the impact of location on these changes.

## 2. Materials and Methods

### 2.1. Design and Sampling

A random sample of adults from three continents were recruited to complete a cross-sectional online survey from May to June 2020. To gain a wide understanding of consumer perceptions, the only inclusion criteria was that participants were aged greater than 18 years. Due to the restrictions of COVID-19, multiple recruitment strategies were undertaken including social media, researcher networks, and an external market research agency (Dynata, London, UK). Participants were recruited from the island of Ireland, Great Britain, New Zealand, and the USA. Due to the nature of the sampling methods, it was not possible to calculate specific response rates. However, across all recruitment strategies 2930 potential participants initially began the survey, with 2360 participants included in the final sample due to incomplete surveys, under the age criteria, or from a different region outside the focus of the study. The participant characteristics can be seen in Table 1. Ethical approval for this study was obtained from the Faculty of Medicine, Health and Life Sciences Research Ethics Committee at Queen’s University Belfast (Reference Number: MHLS20_54). The research was conducted in accordance with the guidelines given in the Declaration of Helsinki. All participants were informed that by taking part in the survey, and they all gave consent for their data to be used.

### 2.2. Procedure

This research is reported in line with the Strengthening the Reporting of Observational studies in Epidemiology (STROBE) Statement [32]. The survey was administered online using SurveyMonkey, with potential participants gaining access through a link in the advertisements or provided by the market research agency. Following information on the survey and consent, participants were screened for eligibility. The survey took approximately 15 min to complete. Sociodemographic information such as age, gender, and education level were obtained.

### 2.3. Survey Measures

The survey was developed and adapted from existing measures and critically reviewed by the multidisciplinary research team for relevance and suitability. Prior to data collection, the questionnaire was piloted with six individuals for clarity and timing, with minor amendments made to the wording. Areas included in the survey encompassed consumer perceptions around cooking skills confidence, changes in meal preparation and food practices, as well as changes in diet quality during the COVID-19 pandemic.

#### 2.3.1. Cooking Related Variables

Due to the extraordinary nature of the COVID-19 pandemic, previously used items [6,7] were critically reviewed by the research team and adapted to ask about “before” the COVID-19 pandemic social isolation and “currently during” the COVID-19 pandemic social isolation. Participants were asked “In a typical week, how often DID/DO you…” about a number of items including: “eat take-away foods or fast-food which are ready to eat as your main meal? (like Chinese, fish and chips or McDonald’s etc.)”; “Throw away food because it goes past its use-by or best before date?”; “To prepare your dinner, Use only fresh, basic or raw ingredients” etc. Before analysis, all items were reverse coded, so that a higher score indicated a higher frequency.

#### 2.3.2. COVID-19 Related Issues

Prevalent consumer factors were assessed in similar “before” and “currently during” the COVID-19 pandemic social isolation questions. Participants were asked about their behaviour using the items: “have difficulty finding the ingredients you were looking for”, and “Buy larger quantities of food products than needed before your next shop (bulk buying)? e.g., flour, rice, pasta”. Participants responded on a five-point Likert scale from “Never” to “every time”.

#### 2.3.3. Food Practices

As no validated measures exist relating to food practices in an emergency situation such as the COVID-19 pandemic, the food skills confidence measure [33] was adapted to a five-point Likert scale ranging from “never” to “every time”. Twelve adapted items from the original food skills confidence measure were included: “plan meals ahead or plan to buy? (e.g., for the day/week ahead)”, “cook more or double recipes which can be used for another meal or freezing”. A further two COVID-19 specific food practices were included after consensus from the research team, including the “bulk buying” in Section 2.3.2 and the item “substitute an ingredient in a recipe due to ingredients not being available”. The sample of participants was randomly split to conduct an exploratory factor analysis (EFA) and confirmatory factor analysis (CFA) to assess the validity of the adapted measure.

#### 2.3.4. Diet Quality

An adaption of the Dietary Instrument for Nutrition Education (DINE) [34], a food frequency questionnaire, was used to assess saturated fat intake and fruit and vegetable consumption as indicators of diet quality. Again, participants were asked about before and during the COVID-19 pandemic, in a typical week, how often they ate or eat a serving of a range of foods on a five-point scale from “None”, to “5 or more times a week”. For fruit and vegetable consumption, participants were asked to report the number of portions of each they consumed per day, with examples provided of what was considered a portion.

### 2.4. Statistical Analysis

Data was analyzed using IBM SPSS v26 and AMOS v25 (SPSS Inc., Chicago, IL, USA). Descriptive statistics (Mean, standard deviation (SD) percentages) were used for demographic data. For the validation of the adapted food practices measure, EFA (maximum likelihood) with direct oblimin rotation was used, as it was assumed that factors would be related [35]. The Kaiser–Meyer–Olkin (KMO) and Bartlett’s Test of Sphericity values were used to assess sample adequacy [36,37]. Factors were assessed using Eigenvalues greater than 1 [38], and a minimum of three items per factor [39]. Based on communalities and factor loadings, items were removed. The final model identified by the EFA was assessed as a confirmatory factor analysis with maximum-likelihood estimation, using IBM SPSS Amos v25. The Root Mean Square Error of Approximation (RMSEA) (preferred value of 0.05 or less) and Comparative Fit Index (CFI), Normed-Fit Index (NFI), Tucker–Lewis Index (TLI) (value of 0.90 or greater acceptable) were used to assess model fit [40].

Intra-region differences were assessed using Wilcoxon signed ranks tests with Bonferroni corrections. Inter region differences were assessed using Welch Analysis of Variance (ANOVA) and Games–Howell post hoc analysis with Bonferroni corrections.

## 3. Results

### 3.1. Participant Sociodemographic Characteristics

Overall, 2360 individuals participated. The sociodemographic characteristics of the sample of each country are presented in Table 1. Participants age ranges were as follows 18 to 79 years (IOI), 18 to 91 years (GB), 18 to 92 years (USA), and 18 to 88 years (NZ). The mean age of the participants ranged from 35.91 on the IOI to 53.69 in the USA. There was a large female majority in the IOI sample (87.5%) with a more equal split in the other regions.

### 3.2. EFA and CFA Validation of Emergency Situation Food Practices Measure

#### 3.2.1. EFA

The results indicated that the sample was adequate for analysis with a KMO value of 0.76, and a significant Bartlett’s Test of Sphericity (*p* < 0.001). The items “follow recipes when cooking”, “compare prices before you buy food”, “know what budget you have to spend on food”, “read the storage and use-by or best before date on food packets” and “Buy larger quantities of food products than needed before your next shop (bulk buying)? e.g., flour, rice, pasta” were removed due to communalities and low factor loadings. A two-factor structure was apparent in the data, as shown by Eigenvalues greater than 1. Both factors had a minimum of three items, no items cross-loaded on more than one factor, and the minimum factor loading was 0.4.

#### 3.2.2. CFA

**Figure 1:**
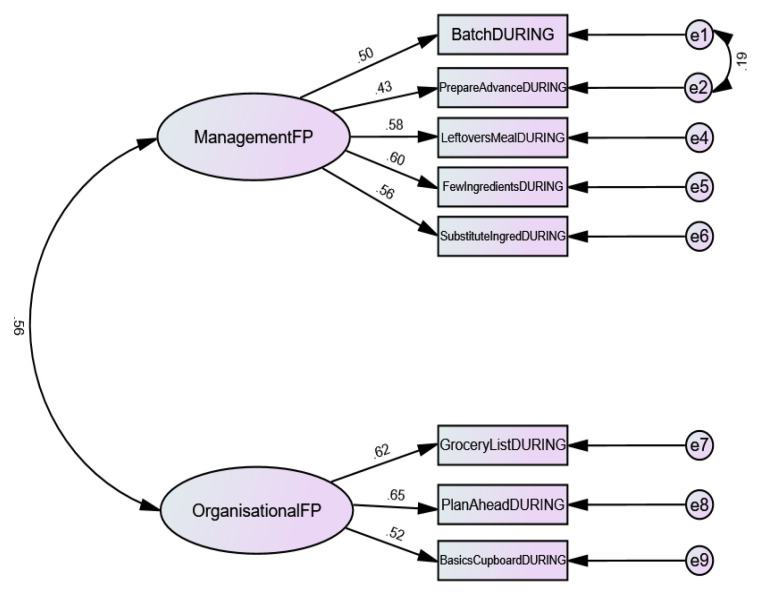
Final measurement model for Emergency Situation Food Practices with standardized factor loadings and correlations. FP: Food Practices; e1–9: error term for each variable.

When entered as a CFA, the final EFA model did not have optimal fit and “Limited time” did not load sufficiently so was removed. Following these amendments, fit was acceptable. Specifically, RMSEA was 0.05. The NFI was 0.95, the CFI was 0.96, and TLI was 0.93, indicating overall acceptable fit. All standardised loadings were 0.40 or above. See Figure 1 below.

### 3.3. Food Practice Changes Relating to COVID-19 within Countries

Examination of the data found that there were changes in practices during COVID-19, compared with before COVID-19. These differences are outlined below, and full differences can be found in Table 2.

#### 3.3.1. Cooking Related Variables

IOI and GB had significant increases in preparing dinners using basic or fresh ingredients (*p* < 0.001), while IOI, GB, and NZ also had a significant reduction in preparing a dinner by buying readymade meals (*p* < 0.001). All four regions had a reduction in the consumption of takeaway (*p* < 0.001). IOI, GB, and NZ had a significant reduction in food waste, from throwing away food (*p* < 0.001), and had a significant increase in baking (*p* < 0.001).

#### 3.3.2. COVID-19 Related Issues

All four regions had a significant increase in difficulty finding ingredients (*p* < 0.001) and a significant increase in bulk buying of ingredients (*p* < 0.001).

#### 3.3.3. Food Practices

All four regions significantly increased their organisational food practices (*p* < 0.001). GB and USA significantly increased their management food practices (*p* < 0.001). For the overall Emergency Situation Food Practices measure, IOI, GB, NZ, and the USA all had significant increases (*p* < 0.001).

#### 3.3.4. Diet Quality

GB significantly increased their consumption of fruit (*p* < 0.001), while IOI, GB, and NZ significantly increased their intake of vegetables (*p* < 0.001). However, IOI, GB, and NZ all also had significant increases in saturated fat intake (*p* < 0.001).

### 3.4. Inter Region Differences

In addition to differences within regions relating to COVID-19, there were also differences apparent between regions. All inter region differences can be found in Table 3.

#### 3.4.1. Cooking Related Variables

IOI had a significantly greater reduction in preparing dinners using readymade meals (*p* < 0.001) and a significantly greater increase in using fresh or basic ingredients in dinner preparations in comparison to the other three regions (*p* < 0.001). There were also significant differences in the changes in eating takeaway foods. Both NZ and IOI saw significantly greater decreases compared with all other countries. IOI significantly reduced their food waste in comparison to the other three regions (*p* < 0.001). In terms of baking, IOI had a significantly greater increase compared with all other regions. GB and NZ saw similar increases, greater than that in the USA (*p* < 0.001).

#### 3.4.2. COVID-19 Related Issues

The increase in difficulty in finding ingredients was significantly greater for those living in both IOI and GB compared with all other regions (*p* < 0.001). There were similar increases in bulk buying for IOI and the USA (*p* < 0.001). While NZ saw the smallest increase in bulk buying, this was similar to the increase in GB.

#### 3.4.3. Food Practices

The increase in organisational food practices was significantly greater for the IOI in comparison to all other regions (*p* < 0.001). There was a similar increase between GB and NZ, as there was between NZ and the USA. For management food practices, GB and the USA saw similar notable increases, while IOI and NZ saw similar (although lower) increases. IOI saw the greatest increase in Emergency Situation food practices, significantly greater than the increase in NZ (*p* < 0.01).

#### 3.4.4. Diet Quality

There were no significant differences in changes in fruit and vegetable consumption between the regions. However, the increase for saturated fat intake was higher for IOI compared to the other regions (*p* < 0.001).

## 4. Discussion

To the best of our knowledge, there are currently no published studies that conducted a cross-continental comparison of changes in consumers’ food practices during the COVID-19 pandemic. This was investigated using, where possible, validated and adapted validated measures. Across all regions there were several changes in consumer practices, with less changes for the USA, which may have been expected due to less dramatic societal shifts due to less restrictions. Additionally, this study highlighted differences between the regions, an important aspect to consider, due to all regions having a similar pre-COVID-19 concern around a decline in cooking and increases in the consumption of convenience food products [15,16,17,18,19,20,21,22].

Due to the nature of the pandemic and sampling limitations, some country sociodemographic characteristics may have influenced the results. The IOI sample is younger, female dominated, and highly educated. Despite a decrease in discrepancies in gender for household chores, cooking is still associated as a female chore [7,10,41] and with a higher education [7,41], and thus less change may be expected in the IOI sample. However, older individuals have been associated with more frequent home cooking [7,41]. Although, these differences may be expected, the IOI patterns were similar to GB and NZ who had similar levels of restrictions, one could be considered stricter and one slightly less strict. Additionally, there were some differences in unemployment levels. Both the UK and the Republic of Ireland instigated temporary unemployment social welfare schemes for those affected by COVID-19 [42], New Zealand was exiting lockdown at data collection, while the majority of USA respondents received a single payment of $1200. This meant IOI and GB respondents were more likely to be classed as temporarily unemployed. The decentralized nature of the USA’s COVID-19 measures [28] may have contributed to the lack of change in consumers’ cooking related variables and diet quality found in this study. If consumers were not switched to a “working from home” model, they may have not spent additional time in the kitchen, and therefore had few changes to their meal preparation. This, in turn, may have contributed to little changes in diet quality if they have not changed their preparation and consumption patterns. However, changes in consumers COVID-19 related practices, e.g., having difficulty finding ingredients and bulk buying, and emergency situation food practices, including organisational and management, were seen alongside the other regions. These behaviours have been seen previously in “shock” events such as earthquakes [43], however, the widespread media and instant connectivity of social media coverage may have intensified psychological responses such as coping mechanisms for stressful unmet situations, easing of anxiety to maintain routine, loss of control, and conforming to social pressure [44,45,46]. While there were some region differences, with the widespread media coverage across all regions, it is understandable to see these changes within all the regions. Additionally, it seems logical that consumers may experience difficulties finding ingredients if there is an increase in bulk buying. However, these practices cause extra pressure on the food system that is at maximum capacity to maintain normal food product flow with labor shortages and movement restrictions [5]. It may be that local food systems need to supplement global food supply chains to maintain normal product flow during emergency times [47]. Consumers have a responsibility to be considerate and only purchase their requirements for a week to 2-week period and to reduce the number of shopping trips, which in turn highlights the importance of organisational food practices during these times.

The two regions that had stricter or more rapidly introduced restrictions, IOI and NZ, did not see an increase in management food practices, in comparison to GB and USA. This may be due to practices such as preparing food in advance and batch cooking, which may be seen as advantageous when time is limited, whereas restricted movements and working from home may limit the extent to which these are required. While these practices may be required for meal preparation behaviours in a time-scarce environment [6], they may not be a high priority in this sort of emergency environment. Additionally, the increase in time available, may have contributed to the shift in cooking practices away from ready to eat dinners to increases in cooking using basic or fresh ingredients, whereas limited time is a factor in convenience food consumption [19,48].

While positive practices were found in the cooking-related variables, such as the increase in cooking with fresh or basic ingredients and the reduction in the use of takeaways, it must be noted that increases in saturated fat intake were also found. While some positive changes were seen in fruit and vegetable intake, in light of the impact of COVID-19 on physical and mental wellbeing [49,50,51,52], increases in saturated fat are essential to consider. Home cooking has been associated with positive health outcomes [10,11], however, what is being cooked is important to understand. One popular source for recipes, and additionally a likely source if confined to your home, the internet, can be a source of less healthy recipes [53]. In addition, there were increases in baking, which positively may have been used to fill increased leisure time, or to learn and demonstrate new skills [54]. While there may be some argument for treat food helping mental health in this type of situation, it is important to maintain a balanced diet, especially with limitations on normal physical activity levels [55]. From a health perspective, the reduction in consumption of takeaways can be seen as positive, however, it may have a negative impact on local economies. Some public houses, restaurants, and hotels have experienced dramatic shifts to their business model and made innovative rapid transitions to delivery services to be financially sustainable [56]. While some of these businesses have, additionally, attempted to help support their local community [57]. In light of this, treating oneself, reciprocating and supporting local eateries, may be an economic and community response to such unprecedented times rather than a health or food response.

### 4.1. Implications for Health and Food Systems for Continued (Local or Regional) Lockdowns and into the Future

This study has important implications for future or continued lockdowns and restrictions related to COVID-19 or future emergency situations. To ensure the resiliency of the food supply chain, restrictions on quantities of certain products may be needed. While some supplementation may be needed from local food supply chains, the general public need reassurance from all levels (from governmental to retail) that the food supply chain can cope with the added pressure. Consumers additionally have a responsibility to limit their purchases to their short-term requirements and not buy in bulk. This can be implemented by using organisational food practices, such as planning ahead and shopping with a grocery list.

Additionally, for maintaining physical and mental health, the importance of consuming a balanced diet, must be emphasized. Continued restrictions, isolation, lockdowns and loss of employment will cause severe emotional distress and add immense pressure to individuals’ mental health [51], further exasperated by physical inactivity [58], that will have long-term societal consequences. These can also contribute to weight gain, an extra concern during COVID-19. This is vital, as obesity has been shown to be a risk for mortality from COVID-19 [59]. Some increases in fruit and vegetable consumption and reduction in takeaway foods were found in this study, which can have a positive impact on mental health and weight maintenance [10,12,14]. Health promotion messages should target these behaviours, as it has been shown they can change in lockdown situations and highlight limiting the consumption of takeaway food. From an economical and health perspective, targeted messages could emphasize the limited consumption of takeaways, however, when consuming these type foods as a treat, it is a good opportunity to support local businesses. Learning and using cooking or baking skills is a valuable use of newfound extra (leisure) time, however, attention must be given to what is being prepared. The promotion of exciting healthy recipes, again a key area to increase fruit and vegetable intake, to try during lockdown is recommended. Further tailoring of recipes to local or seasonable ingredients could be used as a method for supporting local businesses. An additional element for consideration, while not the focus of this study, however as the study findings have implications on it, is the increase in food insecurity during the COVID-19 pandemic especially among individuals who may not have experienced this previously [60]. With the normalization of cooking, promotion of economically friendly recipes that highlight different methods for stretching limited ingredients, and that show methods for using leftovers to create a new meal, should be widely spread throughout all regions. Other budget cookery techniques such as batch cooking or home freezing and using cheaper alternative ingredients could be used in promotion tactics.

### 4.2. Strengths and Limitations

Strengths of this research include the large sample size and the cross-continental sampling to gather global perspectives and changes in consumers’ practices and to investigate the impact of COVID-19 policies both between and within regions. Another strength of this study was the use of validated measures where possible in the questionnaire and validation of adapted measures, such as the adaption of the food skills confidence measure [33] to increase its applicability in these unprecedented times.

While this study has a number of strengths some limitations must be noted, including, as with most cross-sectional research, it is self-report relying on participant memories, which may impact participant responses. Additionally, the differing sampling strategy used for the IOI sample, predominantly social media recruitment, may have led to some selection bias and the higher number of females in this sample. It should also be noted that the use of an online survey, while necessary during the pandemic, may have biased the sample towards those more competent in using technology and therefore may not be representative to each region. However, differences in demographics for completion of online surveys have been reducing or disappearing entirely [61]. Furthermore, the high technological competence in each region and the great proliferation of technology during the pandemic, including older adults increasing technological competencies due to the pandemic [62], which is seen in the broad age ranges in each region, reduces the risk of this bias having a profound impact on the results. To prevent participant fatigue some related areas were not investigated in this study, such as practices related to food safety and whether with increased cooking was there a difference in food safety practices. Changes in food safety knowledge and practices pre- and post-COVID-19 and their relationship to increased levels of cooking is an interesting area for future research.

## 5. Conclusions

This cross-continental comparison of changes in consumers’ food practices found a large number of changes across different food practices during the COVID-19 pandemic, both within and between regions, including organisational food practices such as meal planning. Slightly less changes were seen in cooking related practices, where no changes were seen in meal preparation, in the USA sample, where the differences in social distancing and stay at home policies may have been influential. Food practices, which may put added pressure on the food system, such as bulk buying, were seen across all regions. Additionally, while positive cooking related practices and increases in fruit and vegetable intake were found, an increase in saturated fat intake was seen. During continued lockdown phases and in future emergency situations, the importance of organisational food practices such as planning ahead and shopping with a grocery list should be emphasized to prevent bulk buying and strain on the food system. Furthermore, with the impact of COVID-19 on physical and mental health, the essentiality of maintaining of a balanced diet should be promoted. As there was an increase in cooking seen, the promotion of recipes may be an optimal strategy for increasing fruit and vegetable intake, supporting local businesses through the use of local and seasonal ingredients, and for furthering food budgets through economically friendly recipes.

## Figures and Tables

**Table 1:** Basic Demographics across the different regions.

Country/Region	Island of Ireland		Great Britain		USA		New Zealand	
Characteristic	Mean	SD	Mean	SD	Mean	SD	Mean	SD
Total (n)	538		961		381		480	
Age	35.91	12.52	50.66	15.34	53.69	18.41	45.71	17.20
Body Mass Index	26.03	5.72	26.43	5.38	29.01	8.36	26.61	6.17
	n	%	n	%	n	%	n	%
Gender								
Male	67	12.5	468	48.7	176	46.1	229	47.7
Female	471	87.5	490	51.0	204	53.4	249	51.9
Other			3	0.3	2	0.5	2	0.4
Education								
None			3	0.3			3	0.6
Primary School	3	0.6	11	1.1	3	0.8	10	2.1
Secondary School	74	13.8	238	24.8	66	17.3	104	21.7
Additional Training	85	15.8	217	22.6	76	19.9	106	22.1
Undergraduate degree	176	32.7	310	32.3	132	34.6	161	33.5
Postgraduate degree	200	37.2	182	18.9	105	27.4	96	20.0
Employment Status								
Full Time	300	55.8	396	41.2	130	34.0	237	49.4
Furloughed or temporarily unemployed	72	13.4	114	11.9	26	6.8	13	2.7
Part time (less than 8 h per week)	17	3.2	22	2.3	20	5.2	21	4.4
Part time (greater than 8 h per week)	74	13.8	88	9.2	17	4.5	51	10.6
Retired	11	2.0	233	24.2	149	39.0	80	16.7
Long term sickness/disability	9	1.7	31	3.2	9	2.4	22	4.6
Unemployed (either seeking or not seeking employment)	55	10.2	77	8.0	31	8.1	56	11.7
Working from Home								
All working hours	232	43.1	301	31.3	102	26.7	84	17.5
Some working hours	49	9.1	69	7.2	21	5.5	68	14.2
Not working from home	110	20.4	136	14.2	43	11.3	156	32.5
Essential Worker Status								
Yes	139	25.8	206	21.4	97	25.4	132	27.5
No	233	43.3	290	30.2	60	15.7	167	34.8
Unsure	20	3.7	10	1.0	10	2.6	10	2.1
Parental Status (Under-16)								
Yes	124	23.0	140	14.6	51	13.4	93	19.4
No	268	49.8	664	69.1	266	69.6	294	61.3

**Table 2:** Within region differences before the COVID-19 pandemic to during it on a number of food related variables and on Emergency Situation Food Practices.

	Island of Ireland (*n* = 538)			Great Britain (*n* = 961)			USA (*n* = 382)			New Zealand (*n* = 480)		
Variables	Before	During	Significance	Before	During	Significance	Before	During	Significance	Before	During	Significance
***Cooking Related variables***	Mean (SD)	Mean (SD)	*p*	Mean (SD)	Mean (SD)	*p*	Mean (SD)	Mean (SD)	*p*	Mean (SD)	Mean (SD)	*p*
Dinner—readymade	1.88 (1.06)	1.52 (0.81)	<0.0001	2.35 (1.13)	2.14 (1.13)	<0.0001	2.62 (1.27)	2.53 (1.35)	0.1011	1.98 (1.09)	1.84 (1.16)	0.0002
Dinner—mixed ingredients	3.61 (1.39)	3.58 (1.58)	0.3403	3.29 (1.25)	3.29 (1.37)	0.9960	3.20 (1.39)	3.20 (1.47)	0.7604	3.25 (1.35)	3.29 (1.49)	0.3373
Dinner—fresh ingredients	4.42 (1.17)	4.77 (1.11)	<0.0001	4.12 (1.26)	4.25 (1.37)	<0.0001	3.53 (1.45)	3.55 (1.49)	0.6751	4.29 (1.25)	4.32 (1.35)	0.2351
Eat Take Away	2.58 (0.91)	1.87 (.86)	<0.0001	2.34 (0.98)	1.83 (1.02)	<0.0001	3.01 (1.26)	2.62 (1.37)	<0.0001	2.63 (0.97)	1.55 (1.02)	<0.0001
Throw Away food—prepare too much	2.63 (1.40)	2.14 (1.14)	<0.0001	1.98 (1.20)	1.80 (1.07)	<0.0001	2.11 (1.39)	1.98 (1.33)	0.0042	1.89 (1.14)	1.71 (1.07)	<0.0001
Throw awaypast the use-by date	2.74 (1.04)	2.22 (0.92)	<0.0001	2.06 (1.00)	1.90 (0.99)	<0.0001	2.35 (1.27)	2.20 (1.27)	0.0010	2.01 (0.97)	1.85 (1.01)	<0.0001
Bake	1.92 (0.90)	2.82 (1.17)	<0.0001	2.15 (1.09)	2.45 (1.23)	<0.0001	2.45 (1.34)	2.53 (1.34)	0.0929	2.35 (1.06)	2.74 (1.23)	<0.0001
***COVID-19 related issues***												
Difficulty finding ingredients	2.12 (0.70)	3.08 (0.79)	<0.0001	2.13 (0.78)	2.85 (0.83)	<0.0001	2.25 (0.97)	2.69 (1.03)	<0.0001	2.29 (0.83)	2.70 (0.95)	<0.0001
Bulk buying	2.31 (0.99)	2.92 (1.05)	<0.0001	2.33 (0.98)	2.73 (1.04)	<0.0001	2.63 (1.06)	3.14 (1.14)	<0.0001	2.49 (0.92)	2.82 (1.03)	<0.0001
***Food Practices (FP)***												
Organisational	11.07 (2.49)	12.82 (1.97)	<0.0001	10.95 (2.54)	11.82 (2.48)	<0.0001	10.59 (2.69)	11.18 (2.70)	<0.0001	10.90 (2.45)	11.68 (2.48)	<0.0001
Management	14.78 (2.98)	14.82 (3.04)	0.4379	13.53 (2.99)	13.95 (3.23)	<0.0001	13.27 (3.55)	13.96 (3.90)	<0.0001	14.17 (2.88)	14.27 (3.01)	0.1117
Overall Emergency Situation FP	25.85 (4.63)	27.64 (4.18)	<0.0001	24.48 (4.41)	25.77 (4.62)	<0.0001	23.86 (5.08)	25.14 (5.46)	<0.0001	25.07 (4.34)	25.94 (4.46)	<0.0001
***Diet Quality Indicators***												
Portions of Fruit per day	2.21 (1.32)	2.29 (1.46)	0.0343	2.29 (1.52)	2.39 (1.53)	<0.0001	1.93 (1.60)	2.06 (1.80)	0.0163	2.17 (1.59)	2.30 (1.62)	0.0029
Portions of Veg per day	2.56 (1.37)	2.81 (1.47)	<0.0001	2.66 (1.47)	2.89 (1.63)	<0.0001	2.08 (1.55)	2.29 (1.76)	0.0106	2.62 (1.56)	2.82 (1.71)	<0.0001
Saturated Fat	11.96 (4.57)	13.34 (4.86)	<0.0001	9.82 (4.28)	10.46 (4.58)	<0.0001	10.57 (5.26)	10.89 (5.33)	0.0222	9.98 (4.30)	10.79 (4.82)	<0.0001

Bonferroni correction equals significance level at <0.0008. Cooking related variables were measured on a scale: “Everyday”, “4–6 times a week”, “2–3 times a week”, “Once a week”, “1–2 month”, “Never”, and were reverse coded so that “Everyday” was the highest score. COVID-19 related variables were scored on a 5-point scale, with 1 meaning “Never”, and 5 meaning “Every time”. For Food Practices possible ranges: Organisational 3–15; Management 5–25; Overall Emergency Situation: 8–40. Saturated Fat intake ranged from 0 to 36.

**Table 3:** Inter region differences on changes in food related variables and Emergency Situation Food Practices.

	IOI	GB	USA	NZ	Significance
Change	Mean (SD)	Mean (SD)	Mean (SD)	Mean (SD)	*p*
***Cooking related variables***					
Dinner—readymade	−0.36 (0.89) ^a^	−0.22 (0.85) ^b^	−0.09 (0.97) ^b^	−0.14 (0.89) ^b^	0.000
Dinner—mixed ingred.	−0.03 (0.99)	0.00 (0.77)	0.01 (0.93)	0.05 (0.91)	0.647
Dinner—fresh	0.35 (0.85) ^a^	0.12 (0.75) ^b^	0.02 (0.95) ^b^	0.03 (0.81) ^b^	0.000
Eat Take Away	−0.71 (1.00) ^a^	−0.51 (0.91) ^b^	−0.40 (1.32) ^b^	−1.08 (1.08) ^c^	0.000
Throw Away—too much	−0.49 (0.98) ^a^	−0.18 (0.74) ^b^	−0.12 (0.94) ^b^	−0.18 (0.88) ^b^	0.000
Throw away—date	−0.53 (0.95) ^a^	−0.16 (0.71) ^b^	−0.15 (0.86) ^b^	−0.17 (0.82) ^b^	0.000
Bake	0.90 (0.98) ^a^	0.30 (0.87) ^b^	0.07 (1.01) ^c^	0.39 (0.95) ^b^	0.000
***COVID-19 related issues***					
Difficulty finding ingredients	0.96 (0.98) ^a^	0.72 (0.99) ^b^	0.44 (1.05) ^c^	0.41 (0.92) ^c^	0.000
Bulk buying	0.61 (1.05) ^a^	0.40 (1.01) ^bc^	0.51 (1.12) ^ab^	0.33 (0.89) ^c^	0.000
***Food Practices (FP)***					
Organisational	1.75 (2.39) ^a^	0.87 (1.83) ^b^	0.59 (1.84) ^c^	0.78 (1.73) ^bc^	0.000
Management	0.05 (2.85) ^a^	0.42 (2.15) ^b^	0.69 (2.32) ^b^	0.10 (2.12) ^a^	0.000
Overall Emergency Situation FP	1.79 (4.51) ^a^	1.29 (3.27) ^ab^	1.28 (3.51) ^ab^	0.88 (3.01) ^b^	0.002
***Diet Quality indicators***					
Fruit	0.10 (1.17)	0.13 (1.01)	0.13 (1.24)	0.13 (0.98)	0.950
Veg	0.25 (0.97)	0.24 (0.92)	0.19 (1.16)	0.16 (0.88)	0.391
Saturated Fat	1.40 (3.51) ^a^	0.66 (3.05) ^b^	0.39 (3.57) ^b^	0.83 (3.29) ^b^	0.000

Superscript letters (^a, b, c^) indicate where difference lie between the groups; Bonferroni correction equals significance level at *p* < 0.003.

## Data Availability

The data presented in this study are available on request from the corresponding author. The data are not publicly available due to the ethical approval received.
